# Dexamethasone intravitreal implant for macular edema and some other rare indications in uveitis

**DOI:** 10.3892/mi.2023.99

**Published:** 2023-07-19

**Authors:** Seher Koksaldi, Mustafa Kayabaşi, Zıya Ayhan, Mahmut Kaya, Taylan öztürk, Aylın Yaman, Ali Osman Saatci

**Affiliations:** 1 Department of Ophthalmology, Dokuz Eylul University, 35330 Izmir, Turkey; 2 Mahmut Kaya Eye Clinic, 35330 Izmir, Turkey

**Keywords:** dexamethasone implant, infectious uveitis, macular edema, non-infectious uveitis, phacoemulsification surgery

## Abstract

In the present study, 110 eyes of 81 patients with uveitis who underwent intravitreal dexamethasone implant (IDI) injection and had a follow-up of at least 6 months between January, 2012 and September, 2022, were retrospectively analyzed. A total of 298 IDI injections were administered (mean, 2.71±2.37; range, 1-12). The mean age of the patients was 49.44±16.67 years (range, 15-86 years). The mean follow-up time after the first IDI was 34.31±26.53 months (range, 6-115 months). In total, 77 (95.1%) patients had non-infectious uveitis, while 4 patients (4.9%) received IDI for uveitic macular edema in association with infectious uveitis (1 patient with acute retinal necrosis and 3 patients with systemic tuberculosis). IDI was injected under the umbrella of intravitreal ganciclovir injection in the patient with healed acute retinal necrosis for the associated pseudophakic cystoid macular edema. A total of 6 patients (7.4%) received IDI prior to phacoemulsification surgery to control the possible post-operative macular edema. In addition, 3 patients (3.7%) with Vogt-Koyanagi-Harada disease received bilateral IDI as the systemic therapy could not be administered due to side-effects of the systemic treatment. In total, 1 patient (1.2%) with idiopathic retinal vasculitis, aneurysms and neuroretinitis was treated with IDI injections in both eyes in addition to systemic therapy to reduce the ongoing inflammation. Of note, two eyes (1.8%) received simultaneous single IDI and anti-vascular endothelial growth factor administration for the treatment of unilateral extrafoveal macular neovascularization (one with active serpiginous choroiditis and one with sympathetic ophthalmia). IDI was administered for the treatment of uveitic macular edema in 68 patients (83.9%). Best-corrected visual acuity improved from 0.69±0.64 to 0.60±0.76 logMAR at the final visit (P=0.008). Baseline mean central macular thickness (CMT) was 499.74±229.60 µm (range, 187-1,187 µm) and the mean final CMT was 296.60±152.02 µm (range, 126-848 µm). Intraocular pressure elevation requiring topical antiglaucomatous eye drops occurred in 28 eyes (25.5%). During the follow-up period, bilateral glaucoma surgery was required in 1 patient (1.2%) and 25 of 65 phakic eyes (38.4%) underwent phacoemulsification. Retinal detachment occurred in one eye (0.9%), endophthalmitis in one eye (0.9%), and transient intravitreal hemorrhage occurred in three eyes (2.7%) after the IDI injections. On the whole, the present study demonstrates that although IDI is mostly employed in non-infectious uveitic eyes with macular edema, it can also be administered in cases with systemic therapy intolerance, pseudophakic macular edema prophylaxis, and with great caution, in selected cases involving infectious uveitis and macular edema.

## Introduction

Corticosteroids have been used in the treatment of macular edema due to various retinal diseases, including diabetic retinopathy, retinal vascular occlusion, non-infectious posterior uveitis, and pseudophakic cystoid macular edema (1). In the study by Rajesh *et al* (1), 6,015 intravitreal dexamethasone implant (IDI) injections were administered in various clinical entities, such as diabetic macular edema, retinal vascular occlusion, post-surgical macular edema, uveitis, age-related macular degeneration, macular edema due to tumors and hereditary retinal disorders such as retinitis pigmentosa, an epiretinal membrane with cystoid macular edema, macular neovascularization (MNV) associated with central serous retinopathy, macular telangiectasia, Coats disease with macular edema, MNV due to angioid streaks refractory to anti-vascular endothelial growth factor (VEGF), radiation retinopathy and Purtscher's retinopathy.

The HURON study (2) demonstrated that a single dose (0.7 mg) of the IDI was well tolerated and provided significant improvement in visual acuity and intraocular inflammation that persisted for 6 months in patients with non-infectious intermediate or posterior uveitis (2). Thus, IDI was approved by the United States Food and Drug Administration for the intravitreal treatment of non-infectious uveitis affecting the posterior segment of the eye in 2011 (3,4).

Although the efficacy of IDI has been demonstrated in the treatment of non-infectious type uveitic macular edema (5,6), the present study aimed to demonstrate the rarer indications for IDI administration for the treatment of uveitis apart from macular edema.

## Patients and methods

### Ethics approval

The present retrospective study included patients with uveitis who were treated with IDI injection(s) between January, 2012 and September, 2022 at the Department of Ophthalmology Dokuz Eylul University. Written informed consent was obtained prior to each injection. The approval of the Ethics Committee of Dokuz Eylul University (2022/39-15) was attained for the present study.

### Patients and treatment

A total of 110 eyes from 81 patients who received at least one IDI treatment and had a follow-up of at least 6 months were included in the present study. Uveitic entities were classified according to the updated Standardization of Uveitis Nomenclature (SUN) classifications (7-10). The diagnosis of idiopathic retinal vasculitis, aneurysms and neuroretinitis (IRVAN) syndrome was established according to the criteria set by Samuel *et al* (11). Patients who received any periocular or intravitreal therapy within the past 3 months, pregnant or breastfeeding patients, patients with advanced glaucoma, patients who were on anticoagulant treatment and patients with rubeosis iridis were excluded.

In addition to demographic data, etiology of the uveitis, indication for the dexamethasone implant, best-corrected visual acuity (BCVA), lens status, intraocular pressure (IOP) rise, and related antiglaucoma treatment, immunosuppressive therapy, central macular thickness (CMT) and several IDI injections were noted. Snellen's visual acuity was assessed prior to the first injection and at the final visit and converted into logMAR for statistical analysis. Intraocular pressure was measured using Goldmann applanation tonometry. Patients who experienced an IOP rise ≥10 mm and/or IOP ≥25 mmHg were treated with antiglaucomatous eye drops. The CMT was measured using optical coherence tomography (Heidelberg Spectralis, Heidelberg Engineering). All patients had undergone appropriate clinical evaluation and laboratory investigations whenever necessary.

The injection was performed under topical anesthesia in adults and general anesthesia in younger patients in the operating room at least 3 min after the 5% povidone-iodine instillation. The injections were administered through the inferotemporal quadrant either 3.5 mm or 4 mm posterior to the limbus depending upon the lens status. A topical antibiotic was prescribed four times a day for 5 days.

### Statistical analysis

Statistical analyses were performed using SPSS Statistics version 28 (IBM Corp.). The results of descriptive analyses are expressed as counts and percentages for categorical variables and as the mean ± standard deviations for quantitative variables. The Kolmogorov-Smirnov and Shapiro-Wilk tests were used to determine whether the data were normally distributed. The Wilcoxon signed-rank test was used to compare the baseline BCVA and CMT vs. the final BCVA and CMT, respectively. A P-value <0.05 was considered to indicate a statistically significant difference.

## Results

### Baseline demographics and characteristics

A total of 110 eyes of 81 patients were included in the present study. The mean age of the patients was 49.44±16.67 years (range, 15-86 years) and 48 of the patients (59.3%) were females. In total, 29 patients (35.8%) had bilateral uveitic involvement. Patients were followed-up for a mean of 34.31±26.53 months (range, 6-115 months). Of the 81, 77 patients (95.1%) had non-infectious uveitis, while 4 patients (4.9%) had a concomitant infectious disease. In addition, 3 patients (3.7%) had systemic tuberculosis-associated posterior uveitis and macular edema was successfully treated with IDI injections in addition to systemic therapy. IDI was injected under the umbrella of intravitreal ganciclovir injections in another patient with healed acute retinal necrosis (ARN) and pseudophakic cystoid macular edema (1.2%). In total, 6 patients (7.4%) (idiopathic panuveitis in two cases, idiopathic posterior uveitis in two cases and Behçet's uveitis in two cases) received IDI prior to phacoemulsification surgery to control the post-operative macular edema. Furthermore, 3 patients (3.7%) with Vogt-Koyanagi-Harada (VKH) disease received bilateral IDI, as systemic therapy and could not be employed due to the adverse effects of the systemic treatment (12). IDI was administered for the maintenance of remission in 2 patients with VKH, and IDI was administered during the active disease phase in the remaining patient. Of note, 1 patient (1.2%) with IRVAN syndrome was treated with IDI injections in both eyes in addition to systemic therapy to reduce the ongoing inflammation. In addition, two eyes (1.8%) were treated with a simultaneous single IDI and anti-VEGF administration for the treatment of unilateral extrafoveal MNV associated with active serpiginous choroiditis and sympathetic ophthalmia. IDI was administered for the treatment of uveitic macular edema in the remaining 68 patients (83.9%) (Table I). At the time of the first implant, 34 patients (42%) were under immunosuppressive therapy; 12 eyes (10.9%) were receiving IOP-lowering medication; 42 eyes (38.2%) had clear crystalline lens, 23 eyes (20.9%) had cataract and 45 eyes (40.9%) were pseudophakic. At baseline, the mean BCVA was 0.69±0.64 logMAR and the mean CMT was 499.74±229.60 µm (range, 187-1,187 µm). The baseline characteristics of the patients and eyes are presented in detail in Tables II and III.

### Outcome of the last visit

**Figure 1:**
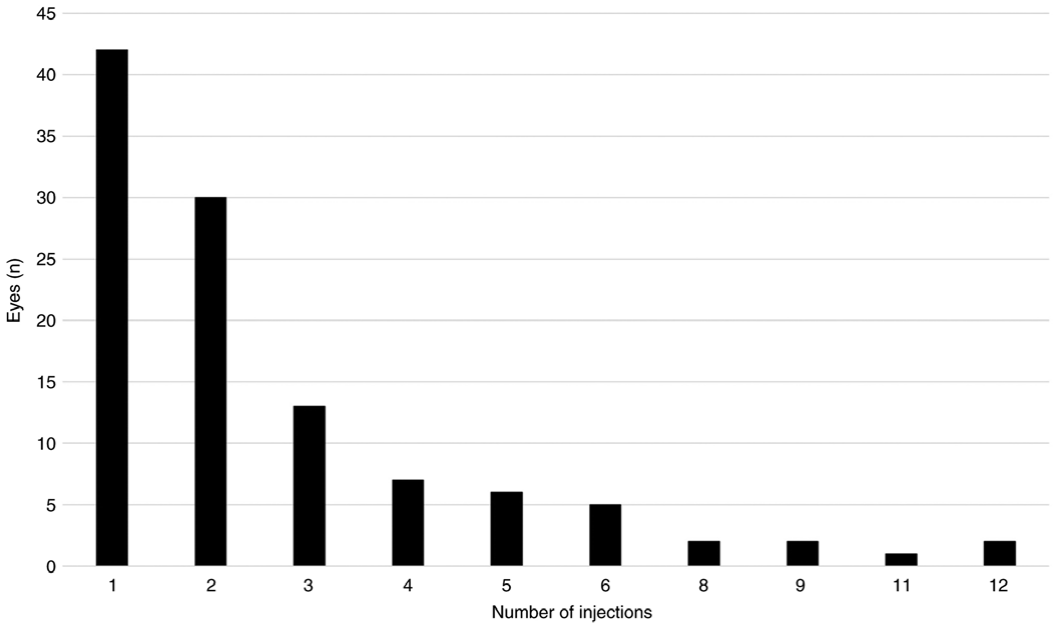
Distribution of the intravitreal dexamethasone implant injection numbers in the patients in the present study.

During the study period, a total of 298 IDI injections were administered. A total of 42 (38.2%) received a single injection, 30 eyes (27.3%) had two injections, and 38 eyes (34.5%) between three and 12 injections (Fig. 1). The mean administered number of IDI injections was 2.71±2.37 (range, 1-12). In 51 eyes (46.4%), BCVA was improved at least for two Snellen lines at the final visit and the mean final BCVA was improved significantly to 0.60±0.76 logMAR (Wilcoxon test, P=0.008). The mean final CMT decreased significantly to 296.60±152.02 µm (range, 126-848 µm) at the last visit (Wilcoxon test, P<0.001) (Table IV). A total of 41 patients (50.6%) were under immunosuppressive therapy at the final visit.

Steroid-induced glaucoma occurred in 28 eyes (25.5%) following a mean of 1.61±1.40 injections (range, 1-6); and 1 patient underwent bilateral glaucoma surgery following a single injection in both eyes. In addition, 25 of the 42 eyes (59.5%) with a clear crystalline lens at the baseline developed cataracts during the follow-up and cataract surgery was performed in 11 eyes. Of the 23 eyes that had already some cataract at the baseline, 14 (60.8%) underwent cataract surgery due to cataract progression. Overall, 25 of 65 phakic eyes (38.4%) underwent cataract surgery during the follow-up. While retinal detachment (RD) occurred following the only injection in one eye (0.9%) transient intravitreal hemorrhage occurred in three eyes (2.7%) following the only injection in a patient's right eye and following the tenth and ninth injections in another patient's right and left eyes, respectively. Endophthalmitis was also noted in a patient (0.9%) following the only injection. The eye with the RD was successfully treated with vitreoretinal surgery. The eye with the endophthalmitis was successfully treated with intravitreal antibiotic injections and subsequent pars plana vitrectomy (Table V).

## Discussion

The treatment of uveitic macular edema can sometimes prove difficult, as the blood-retinal barrier limits the efficacy of medications to suppress the inflammation sufficiently (13). The dexamethasone 0.7 mg implant has been shown to be effective and safe for the local treatment of chronic cystoid macular edema secondary to non-infectious uveitis in previous studies (6,13). While the dexamethasone implant is primarily administered for the treatment of uveitic macular edema, it is also employed in other rare uveitic occasions, such as birdshot chorioretinopathy (14), diffuse uveal melanocytic proliferation (15), serpiginous choroiditis (16), exudative RD associated with uveal melanoma (17), punctate inner choroidopathy (18), autoimmune retinopathy (19), ampiginous choroidopathy, tubulointerstitial nephritis and uveitis syndrome, sympathetic ophthalmia and IRVAN syndrome (20). The studies that included at least 50 uveitic cases who received IDI injections (1,2,20-30) are summarized in Table VI.

Surgery for uveitis-induced cataract can be more troublesome than conventional cataract surgery due to the presence of synechia, pupillary membrane, possible iris hemorrhage and an increased risk of post-operative inflammation that can cause synechia and inflammatory membrane on the anterior surface of the intraocular lens (IOL), early posterior capsular opacification, and macular edema (31). A number of surgeons prefer to utilize steroids pre-operatively or intraoperatively to improve the visual outcome, reduce post-operative inflammation and prevent subsequent macular edema (31,32). Li *et al* (33) reported 10 eyes of 7 patients with refractory panuveitis (five eyes with VKH, three eyes with idiopathic panuveitis, one eye with sclerouveitis, and one eye with sympathetic ophthalmia) who underwent cataract surgery and combined IDI injection. They compared the change in CMT at the 1-, 3- and 6-month follow-up visits with the pre-operative visit and found that there was no significant change in CMT. In some case reports, the efficacy of an IDI administered prior to cataract surgery was evaluated in patients with uveitic cataracts (34,35). Cordero-Coma *et al* (34) administered IDI 1 month before the cataract surgery in a pediatric patient with chronic anterior uveitis, and reported that the patient tolerated the procedure well and did not exhibit any adverse effects or recurrence in inflammation during a follow-up of 10 months. da Rocha Lima *et al* (35) described a 57-year-old male with HLA-B27-associated chronic uveitis who underwent phacoemulsification and IOL implantation. In the first eye, IDI was injected concurrently with the surgery and there was a severe fibrin inflammatory reaction with 3+ anterior chamber cells on the first post-operative day. The fibrin reaction was completely resolved following the intracameral injection of recombinant tissue plasminogen activator. Intravitreal dexamethasone implant was administered 5 days prior to cataract surgery of the fellow eye and this time, no anterior chamber reaction was observed on the first postoperative day (35). The authors of that study argued that high concentrations of vitreal dexamethasone may be reached several days after the implantation and pre-operative injection of IDI might provide a sufficient dexamethasone concentration to reduce the post-operative inflammation better (35). Concurrently, the pre-operative administration of IDI prior to the phacoemulsification surgery successfully controlled the post-operative inflammation in eight eyes of 6 patients with chronic recurrent uveitis in the present study.

IDI has been used as an alternative treatment modality to control inflammation in patients with active VKH disease where systemic treatment was terminated due to side-effects or where systemic therapy could not fully control the inflammation (12,36). Elhamaky (37) administered IDI in 29 eyes of 16 patients with refractory VKH disease who experienced comorbidity, dependence, or non-compliance with the systemic steroid and/or immunosuppressive agents, and concluded that dexamethasone implant resulted in improvement in visual acuity, a reduction in macular edema and minimized the burden of systemic steroids. The authors have recently reported our experience with IDI in six eyes of 3 patients with VKH disease in whom hepatic dysfunction occurred in association with systemic immunosuppressive therapy and suggested that IDI injections may be beneficial for achieving a recurrence-free follow-up period without any systemic treatment in selected cases (12).

IDI injection has been effectively employed to obtain rapid control of active inflammation and prevent further recurrences and progressive scarring in serpiginous choroiditis (16). The authors previously treated a single eye with an active serpiginous choroiditis complicated by unilateral extrafoveal MNV with a simultaneous single IDI and ranibizumab administration in addition to systemic treatment (38). Similarly, the authors of the present study also administered IDI and simultaneous aflibercept injection in addition to systemic treatment in a case with sympathetic ophthalmia who had moderate vitritis and extrafoveal MNV in addition to ongoing systemic treatment to obtain both inflammation control and treat the MNV.

In a very recent study, the Multicenter Uveitis Steroid Treatment Trial (MUST) Research Group (39) compared the efficacy of intravitreal dexamethasone implant, methotrexate and ranibizumab administration for the treatment of persistent or recurrent uveitic macular edema in 225 eyes of 194 patients. Each treatment modality led to a significant reduction in CMT when compared to baseline at the 12-week visit (35% for the dexamethasone implant, 11% for the methotrexate and 22% for the ranibizumab). However, the reduction in macular edema was notably more prominent in the dexamethasone implant group (P<0.001 vs. methotrexate and P=0.018 vs. ranibizumab) and only IDI treatment led to a statistically significant improvement in BCVA during the follow-up (4.86 letters; P<0.001) (39). However, the authors of that study also emphasized that dexamethasone implant administration was associated with a higher risk of IOP elevation compared to the other two treatment options (39).

Although IDI is administered for non-infectious uveitic macular edema, it can also be employed in selected cases with infectious uveitis. Fonollosa *et al* (40) reviewed the clinical courses in eight eyes of 7 patients with infectious uveitis. ARN was found in 2 cases, syphilitic intermediate uveitis in a single case, brucellosis characterized with posterior uveitis in 1 case, Lyme disease presenting with anterior uveitis and scleritis in 1 case, toxoplasmic panuveitis in 1 case, and cytomegalovirus related anterior uveitis in 1 case. They administered IDI for the treatment of macular edema and all patients either already had appropriate systemic antimicrobial therapy or received systemic antimicrobial treatment concomitantly. Visual acuity was improved in all eyes and none of the eyes had macular edema at the last visit of a follow-up of mean 18 months (40). Hasanreisoglu *et al* (22) presented a tuberculosis-related uveitis case in a 44-year-old woman where ocular inflammation could be partly treated by anti-tuberculosis treatment, systemic methylprednisolone therapy and sub-tenon triamcinolone acetonide injection and inflammation could only be well-controlled with the IDI injection.

In the present study, four eyes of three patients had tuberculous-associated posterior uveitis and macular edema and were successfully treated with IDI injections. Treatment with interferon α-2a has also been shown to be effective in the management of refractory macular edema in non-infectious uveitis and presumed intraocular tuberculosis (41); however, this treatment could not be employed in the present study due to reimbursement issues and unavailability. IDI was also administered under the umbrella of intravitreal ganciclovir injections in one eye of a patient with healed ARN and pseudophakic cystoid macular edema.

As previously demonstrated, the most notable side-effects of IDI injections are an increase in IOP and cataract formation (42). Rajesh *et al* (1) administered a total of 6,015 IDI injections in 2,736 eyes of 1,441 patients and 149 eyes (5.4%) had non-infectious uveitis. They reported a new onset IOP rise in 20.9% of patients and stated that a total of 576 eyes (32.5% of phakic eyes) developed significant cataracts requiring surgical intervention during the follow-up period (1). The present study noted an increase in IOP in only 25.2% of patients, which was adequately managed with antiglaucomatous medications and it was more likely to occur following the first injection. Both eyes of 1 patient required glaucoma drainage device implantation. Although trabeculectomy has been traditionally considered the main surgical approach for glaucoma surgery (43), drainage implant surgery may be preferred, as there is a higher failure rate in uveitic glaucoma eyes with trabeculectomy (44). In the present study, bilateral drainage implant surgery was performed on the patient with tuberculosis-related posterior uveitis. In addition, herein, 25 out of 65 phakic eyes (38.4%) underwent cataract surgery during the follow-up period.

Even though the present study found that at the final visit, there was a statistically significant reduction in the mean CMT, the essential aim was to point out the various indications of intravitreal dexamethasone implant administration in patients with uveitis, apart from the well-established indication for the treatment of uveitic macular edema. The retrospective nature of the present study was the major limitation and in addition, the authors could not analyze the clinical efficacy of dexamethasone implant among the several uveitic entities as there was a vast number of uveitic entities in the present case series, some with only a few patients.

In conclusion, the present study demonstrates that IDI may also enable clinicians on certain other occasions to achieve improved anatomic and visual outcomes with prior administration before cataract surgery in uveitic eyes, in cases where systemic therapy cannot be employed and in some selected cases with infectious uveitis with recalcitrant macular edema.

## Figures and Tables

**Table I:** Treatment indications for dexamethasone implant of patients in the present study.

Indication	No. of patients (%)
Non-infectious uveitic macular edema	65 (80.2)
Tuberculosis-related posterior uveitis and macular edema	3 (3.7)
Pseudophakic cystoid macular edema	
Healed acute retinal necrosis	1 (1.2)
Prior to cataract surgery	
Idiopathic panuveitis	2 (2.4)
Idiopathic posterior uveitis	2 (2.4)
Behcet's disease	2 (2.4)
Due to adverse effects of systemic treatment	
Vogt Koyanagi Harada disease	3 (3.7)
As an addition to ongoing systemic therapy to achieve better inflammation control	
IRVAN syndrome	1 (1.2)
In combination with anti-VEGF injections for the treatment of inflammatory MNV	
Serpiginous choroiditis	1 (1.2)
Sympathetic ophthalmia	1 (1.2)

IRVAN, idiopathic retinal vasculitis, aneurysms, and neuroretinitis; MNV, macular neovascularization; VEGF, vascular endothelial growth factor.

**Table II:** Baseline demographics and clinical features of the 81 patients prior to the first dexamethasone implant administration.

Characteristic	Frequency
Age, years, mean ± SD (range)	49.44±16.67 (15-86)
Sex, female/male	48/33
Bilateral uveitis, n (%)	29 (35.8)
Follow-up duration, months, mean ± SD (range)	34.31±26.53 (6-115)
Etiology, n (%)	
Idiopathic panuveitis	19 (23.5)
Idiopathic posterior uveitis	18 (22.2)
Behcet's uveitis	14 (17.3)
Idiopathic intermediate uveitis	7 (8.6)
Ankylosing spondylitis	6 (7.4)
Juvenile idiopathic arthritis	4 (4.9)
Vogt-Koyanagi-Harada disease	3 (3.7)
Tuberculosis	3 (3.7)
Acute retinal necrosis	1 (1.2)
IRVAN syndrome	1 (1.2)
MEWDS-like reaction	1 (1.2)
Sarcoidosis	1 (1.2)
Multifocal choroiditis	1 (1.2)
Serpiginous choroiditis	1 (1.2)
Sympathetic ophthalmia	1 (1.2)
Concurrent systemic treatment, n (%)	34(42)

IRVAN, idiopathic retinal vasculitis, aneurysms, and neuroretinitis; MEWDS, multiple evanescent white dot syndrome.

**Table III:** Ocular status of the patients at the ocular examination (110 eyes).

Variable	Frequency
BCVA, logMAR, mean ± SD	0.69±0.64
CMT, µm, mean ± SD (range)	499.74±229.60 (187-1,187)
Eyes receiving IOP lowering medication, n (%)	12 (10.9)
Lens status, n (%)	
Clear crystalline lens	42 (38.2)
Cataract	23 (20.9)
Pseudophakic	45 (40.9)

BCVA, best corrected visual acuity; CMT, central macular thickness; IOP, intraocular pressure.

**Table IV:** BCVA and CMT values of the patients at baseline and at the final visit.

	At baseline	At the final visit	P-value
BCVA, logMAR, mean ± SD	0.69±0.64	0.60±0.76	0.008^a^
CMT, µm, mean ± SD (range)	499.74±229.60 (187-1,187)	296.60±152.02 (126-848)	<0.001^a^

^a^Indicates a statistically significant difference (P<0.05), as determined using the Wilcoxon signed-rank test. BCVA, best-corrected visual acuity; CMT, central macular thickness.

**Table V:** Treatment outcomes of the patients at the final visit and treatment-related side-effects.

Variable	Frequency
No. of patients/eyes	81/110
BCVA, logMAR, mean ± SD	0.60±0.76
CMT, µm, mean ± SD (range)	296.60±152.02 (126-848)
Concurrent systemic treatment, no. of patients (%)	41 (50.6)
Adverse event, no. of eyes (%)	
Steroid-induced glaucoma	28 (25.5)
Glaucoma surgery	2 (1.8)
Cataract formation	25 out of 42 (59.5)
Cataract surgery	25 out of 65 (38.4)
Intravitreal hemorrhage	3 (2.7)
Retinal detachment	1 (0.9)
Endophthalmitis	1 (0.9)

BCVA, best -corrected visual acuity; CMT, central macular thickness.

**Table VI:** Studies that included at least 50 uveitic cases with IDI injections.

Authors	No. of eyes/implants	Rise in intraocular pressure	Cataract formation	Other complications	(Refs.)
Rajesh *et al*	149/381 (2,736/6,015 in total)	20.9%	47.1%	Endophthalmitis (0.07%); retinal detachment (0.03%); vitreous hemorrhage (0.03%)	(1)
Lowder *et al*	77/77	23%	15%	Retinal detachment (2.6%)	(2)
Alba-Linero *et al*	79/134	30.3%	NA	Vitreous hemorrhage (0.7%)	(20)
Mathis *et al*	152/358	28.3%	45.1%	NA	(21)
Hasanreisoglu *et al*	62/87	8%	46%	NA	(22)
Zarranz-Ventura *et al*	82/142	40.2%	NA	Endophthalmitis (0.7%); vitreous hemorrhage (2.1%)	(23)
Pohlmann *et al*	109/298	NA	NA	None	(24)
Zeng *et al*	91/130	14.9%	NA	None	(25)
Breitbach *et al*	59/NA	21%	31%	None	(26)
Tufail *et al*	151/NA	14.2%	39%	Endophthalmitis (0.5%); vitreous hemorrhage (2%)	(27)
Thorne *et al*	78/125	3.8%	NA	None	(28)
Bodaghi *et al*	97/190	20.6%	1%	Vitreous hemorrhage (2%)	(29)
Kang *et al*	52/110	NA	NA	NA	(30)
Present study^a^	110/298	25.5%	59.5%	Endophthalmitis (0.9%); retinal detachment (0.9%); vitreous hemorrhage (2.7%)	

^a^Studies included infectious uveitis. NA, not available.

## Data Availability

The datasets used and/or analyzed during the current study are available from the corresponding author on reasonable request.
